# Multidimensional Recovery Trajectories Following Physiotherapy with or Without Pain Education in People with Chronic Low Back Pain

**DOI:** 10.3390/jcm15062320

**Published:** 2026-03-18

**Authors:** Ahmed Alalawi

**Affiliations:** Department of Medical Rehabilitation Sciences, Faculty of Applied Medical Sciences, Umm Al-Qura University, Makkah 24382, Saudi Arabia; amalawi@uqu.edu.sa

**Keywords:** chronic low back pain, recovery trajectories, pain education, physiotherapy, multidimensional outcomes

## Abstract

**Background/Objective:** To investigate short-term multidimensional recovery trajectories after physiotherapy with or without adjunctive pain education in individuals with Chronic low back pain (CLBP). **Methods:** This was a secondary analysis of a randomized controlled trial (RCT) of 92 participants (46 participants per group) comparing physiotherapy alone with physiotherapy plus pain education. Changes from baseline values over six weeks were calculated for pain intensity, disability, psychological well-being, and self-efficacy to define short-term recovery trajectories across domains, and were standardized prior to analysis. Descriptive characterization of recovery dimensions by principal component analysis and identification of different recovery trajectory clusters by k-means clustering were performed. Sensitivity analyses with multinomial logistic regression were performed to determine robustness after adjustment for baseline characteristics. **Results:** Three recovery trajectories were found: minimal recovery (n = 40), psychosocial-dominant recovery (n = 26), and global recovery (n = 26). In the physiotherapy-only group, participants were classified as minimal recovery (61%) or psychosocial-dominant recovery (39%), with no cases of global recovery. In contradistinction, 57% of participants receiving physiotherapy with pain education were classified as within the global recovery trajectory, with fewer classified as minimal recovery (26%) or psychosocial-dominant recovery (17%). Psychosocial-dominant recovery occurred in both groups, and was characterized by large improvements in psychological well-being and self-efficacy with more modest changes in pain and disability. The distribution of recovery trajectories between treatment groups was large (χ^2^(2)= 36.25, *p* < 0.001; Cramer’s V = 0.63). **Conclusions:** Distinct short-term recovery trajectories were found after physiotherapy with or without pain education in individuals with CLBP, reflecting heterogeneity in multidimensional recovery that is not reflected in mean-based outcome analyses.

## 1. Introduction

Low back pain (LBP) represents a major cause of disability worldwide and remains a major challenge for healthcare systems, despite extensive research and development of clinical guidelines [1,2,3]. In addition to the severity of pain, people with chronic low back pain (CLBP) often experience ongoing disability, low psychological well-being, and low confidence in their ability to cope with symptoms, which reflects the multidimensional nature of the condition [4,5]. Although physiotherapy-based interventions are widely recommended and routinely implemented, the effects of these treatments tend to be small, and large variability in individual responses is consistently observed [6,7].

CLBP is largely conceptualized within a biopsychosocial model. According to this model, biological factors interact with psychological and social factors to influence pain and disability [5,8,9]. The purpose of pain education is to change maladaptive beliefs about pain and reduce perceived threat [10]. It also promotes an adaptive understanding of central sensitization and pain mechanisms [10,11,12]. These interventions can affect multiple domains simultaneously by modifying the cognitive appraisal of pain. These domains include psychological well-being, self-efficacy, and functional engagement [13]. Improvements in psychological well-being may enhance motivation and resilience [14]. Increased confidence in symptom management may support activity despite pain [14,15,16]. Recovery may therefore occur as coordinated change across pain and disability and psychological outcomes rather than single measure improvement. Multivariate trajectory approaches may be appropriate to capture these multidimensional patterns of recovery that mean-based analyses may miss.

Despite the multidimensional and heterogeneous nature of LBP, most randomized controlled trials continue to measure treatment effectiveness by mean changes in isolated outcomes (mainly pain intensity or disability). This can be seen in many RCTs where pooled treatment effects are primarily reported as average between-group differences in pain and functional limitation [17,18,19]. While this approach is appropriate for estimating the average treatment effect, it has limited consideration of normal individual variability [20,21,22]. Consequently, it offers limited information on how patients recover individually in several domains of outcomes. Clinically meaningful patterns of improvement, for example, changes that do not occur simultaneously between symptoms and psychological outcomes, may therefore be masked when recovery is summarized with aggregate statistics only [23,24]. This limitation is especially relevant in LBP, in which improvements in coping, self-efficacy, or well-being may be independent of, or precede, improvements in pain and disability [4].

In response to these limitations, an emerging interest has emerged in approaches that characterize recovery as a multidimensional and heterogeneous process rather than a uniform change in a single outcome. Several studies have found subgroups of patients with different recovery profiles, highlighting that individuals may follow different courses despite receiving similar interventions [24,25,26,27]. However, much of this literature has focused on long-term trajectories or single outcome domains, most commonly pain or disability. Recovery trajectories incorporating both clinical and psychosocial outcomes after intervention remain relatively understudied, leaving the immediate post-treatment recovery of patients across multiple domains incompletely understood.

In a recent study, improvements in psychological well-being partially mediated reductions in disability following a program of pain education combined with physiotherapy, whereas self-efficacy did not [28]. Although the study provided insight into possible pathways of change, it did not examine whether patients demonstrated distinct multidimensional recovery trajectories over time. Exploring how outcomes change in parallel across domains may therefore provide insight beyond mediation models that focus on isolated causal pathways.

Therefore, the aim of this study was to identify short-term multidimensional recovery trajectories following physiotherapy with or without pain education in individuals with CLBP. It was hypothesized that different recovery trajectories would be identified across pain, disability, psychological well-being, and self-efficacy, and that the distribution of these patterns would differ between treatment groups.

## 2. Materials and Methods

### 2.1. Study Design

This study is a secondary analysis of a previously published randomized controlled trial (RCT) [13]. The study examined the effect of adding a structured pain education program to physiotherapy in individuals with CLBP [13]. The primary trial was a two-arm, parallel-group trial with random assignment of participants to standard physiotherapy or physiotherapy combined with pain education.

Block randomization (block size = 4) was used. The sequences of allocations were pre-generated and hidden in sealed, opaque envelopes, which were opened sequentially once participants were enrolled. The allocation of treatment was not disclosed to the participants, and the assessments of outcomes were performed by physiotherapists who were blinded to group assignment. Additional details about the study methodology have been reported elsewhere [13].

The trial was conducted in accordance with the Declaration of Helsinki. The study was approved by the Institutional Ethics Committee (RP/114/2021) and registered in advance on the Clinical Trial Registry (CTRI/2021/08/035963). The current study is based on the initial trial [13], with an emphasis on short-term follow-up changes and multivariate analysis of recovery trajectories at the 6-week follow-up.

### 2.2. Participants

Participants were recruited for the original RCT until the target sample size was reached. Adults aged 18 to 60 years with a clinical diagnosis of non-specific low back pain persisting for more than three months were eligible for inclusion. Diagnosis was established by a qualified medical professional based on clinical assessment. Participants were excluded if they failed to attend more than two scheduled intervention sessions or chose to withdraw from the study. Written informed consent was obtained from all participants prior to enrollment. A total of 92 participants were available for analysis, with 46 allocated to the physiotherapy-only (control) group and 46 to the physiotherapy with pain education group.

### 2.3. Interventions

#### 2.3.1. Physiotherapy (Control Group)

Participants in both groups received a standardized physiotherapy intervention for LBP, consistent with current clinical practice guidelines [7,29]. The program included recommendations to stay active, superficial heat application, lumbar stretching, aerobic conditioning via stationary cycling, and core stabilization with a focus on trunk strength and endurance. The sessions were about 40 min long and provided based on a pre-established protocol [13].

#### 2.3.2. Pain Education (Intervention Group)

In addition to the physiotherapy program, participants assigned to the intervention group received a structured pain education program. The program covered the main concepts regarding chronic pain, pain neurophysiology, central sensitization, fear-avoidance, and the role of psychological and social factors in pain perception. Presentation, visual aids, and interactive discussion were used to deliver the educational program.

The intervention included two pain education sessions per week for the first three weeks, with reflective question-and-answer discussions in weeks four and five. A printed pain education handbook was designed specifically for the trial and distributed to participants at the end of the intervention period [10,11,12].

### 2.4. Outcome Measures

Clinical and psychosocial outcomes were measured at baseline and post-intervention (6 weeks). The present analysis focuses on change over this interval in pain intensity, disability, psychological well-being, and self-efficacy. All outcomes were collected by assessors who were trained and blinded to the group allocation.

Pain intensity was measured using a 10-point Visual Analog Scale (VAS), with 0 representing no pain and 10 representing the worst imaginable pain. The VAS is a valid and reliable tool for measuring pain intensity [30]. Disability was measured using the Roland–Morris Disability Questionnaire (RMDQ), which has 24 items with a range of 0 to 24; the higher the score, the greater the disability [31]. The validity and reliability of the RMDQ have been established [31].

The psychological well-being was measured using the World Health Organization Five-Item Well-Being Index (WHO-5) [32]. The instrument comprises five items that measure participants’ current emotional well-being. Item responses are summed to give a raw score between 0 and 25; the lower the score, the poorer the well-being. For interpretability, raw scores were standardized to a 0–100 scale by multiplying the total score by 4, with higher values indicating better psychological well-being. The WHO-5 has good validity, reliability, and responsiveness in both clinical and general populations [32].

The General Self-Efficacy Scale (GSE) was used to measure self-efficacy [33]. GSE is a 10-item self-report instrument that assesses a person’s perceived ability to cope with difficult situations. The range is 10–40, with higher scores indicating greater perceived self-efficacy. The scale has a high construct validity and internal consistency between different populations [33].

### 2.5. Baseline and Clinical Covariates

In addition to the primary outcomes, a set of demographic and clinical variables was collected at baseline to support adjusted and sensitivity analyses. These variables were selected a priori based on their established associations with pain severity, disability, and recovery trajectories in individuals with CLBP [2,4,34].

Age and sex were used as demographic variables. Clinical and lifestyle-related variables included body mass index (BMI), smoking (yes/no), the duration of LBP (in months), and self-reported sleep duration. These variables were not treated as outcomes in the present analysis. Rather, they were applied to (i) determine baseline comparability between treatment groups and (ii) adjust multivariable models in sensitivity analysis of the robustness of recovery trajectory classification and group differences.

### 2.6. Statistical Analysis

Baseline characteristics were summarized by treatment group using means and standard deviations for continuous variables and frequencies with percentages for categorical variables. For each participant, within-participant change scores were calculated as the difference between post-intervention and baseline values for all outcomes: VAS, RMDQ, WHO-5, and GSE. These changes represented the treatment effect over the 6-week intervention period. In the present study, recovery trajectories were defined as short-term change between baseline and post-intervention across multiple outcome domains. For multivariate analyses, the within-participant change scores were standardized to z-scores to ensure comparable scaling across outcomes [35], before dimension reduction and clustering.

#### 2.6.1. Principal Component Analysis

Principal component analysis (PCA) [36] was applied to the standardized within-participant change values of VAS, RMDQ, WHO-5, and GSE to explore underlying dimensions of short-term recovery trajectories across all outcomes. Components were retained based on eigenvalues > 1, the amount of variance explained, or considering the elbow criterion in the scree plot [35,36,37]. Variable loadings were examined to characterize the contribution of each outcome to the derived components [38].

#### 2.6.2. Recovery Trajectory Identification

Recovery trajectory clusters were identified using k-means clustering [39] applied to the standardized multidimensional change-score space. Solutions with two to four clusters were evaluated. The optimal number of clusters was determined based on a combination of within-cluster sum of squares, mean silhouette width, and clinical interpretability of cluster profiles [40].

A three-cluster solution was selected as the final model. Clusters were labeled post hoc according to their mean change profiles across pain, disability, psychological well-being, and self-efficacy, resulting in the following recovery trajectories: minimal recovery, psychosocial-dominant recovery, and global recovery. Each recovery trajectory was summarized using the mean of within-participant change scores, standard deviations, and 95% confidence intervals for all outcomes. These summaries were used to describe the clinical and psychosocial profiles of each trajectory.

#### 2.6.3. Group Comparisons of Recovery Trajectories

Differences in the distribution of recovery trajectories between treatment groups were assessed using a chi-square test of independence. Effect size was quantified using Cramer’s V. For comparisons involving sparse cells, particularly when examining the presence versus absence of global recovery, Fisher’s exact test was used in place of the chi-square test.

#### 2.6.4. Sensitivity Analyses

Additional sensitivity analyses using multinomial logistic regression [41] were undertaken to assess the robustness of recovery trajectory cluster classification after adjustment for baseline characteristics (e.g., age, sex, duration of LBP, baseline pain and disability, and smoking status). Detailed results are provided in the Supplementary Materials. Analyses were performed in R software (version 4.5.2) and conducted using complete-case data.

## 3. Results

### 3.1. Participants and Baseline Characteristics

Ninety-two participants were included in the analyses, with 46 allocated to the control group and 46 to the pain education group. Baseline data were complete for all reported variables (Table 1). Groups were broadly comparable at baseline: age, pain intensity, well-being, self-efficacy, body mass index, and hours of sleep all showed small standardized mean differences (SMDs ≤ 0.27). More substantial imbalances were observed for the duration of LBP (35.85 [50.37] vs. 20.93 [34.33] months; SMD = −0.35), with longer symptom duration in the control group. The pain education group also included a higher proportion of smokers (26.1% vs. 8.7%).

### 3.2. Recovery Dimensions and Trajectory Cluster Identification

Clustering of multidimensional change scores identified three distinct recovery trajectories. The three recovery trajectories are summarized in Table 2. The minimal recovery trajectory (n = 40) was characterized by small average improvements across all outcomes: pain intensity decreased by −0.83 (SD 1.77; 95% CI: [−1.39]–[−0.26]), disability decreased by −1.58 (SD 3.20; 95% CI [−2.60]–[−0.55]), and gains in well-being and self-efficacy were modest (WHO-5 2.50 [SD: 7.62]; 95% CI 0.06–4.94; GSE 0.75 [SD: 2.22]; 95% CI 0.04–1.46).

The psychosocial-dominant recovery trajectory (n = 26) showed a different profile: pain and disability improved only modestly (VAS −1.54 [SD: 1.58]; 95% CI [−2.18]–[−0.90]; RMDQ −1.73 [SD: 2.16], 95% CI [−2.60]–[−0.86]), whereas self-efficacy and well-being increased substantially (GSE 9.46 [SD: 2.75], 95% CI 8.35–10.57) and well-being (WHO-5 4.92 [SD: 7.03], 95% CI 2.09–7.76).

The global recovery trajectory (n = 26) showed large and coherent improvements across domains, with substantial reductions in pain (VAS −4.00 [SD: 1.30], 95% CI [−4.52]–[−3.48]) and disability (RMDQ −9.77 [SD: 3.44], 95% CI [−11.16]–[−8.38]), accompanied by marked increases in well-being (WHO-5 24.19 [SD: 12.28], 95% CI 19.23–29.15) and moderate gains in self-efficacy (GSE 2.69 [SD: 4.36], 95% CI 0.93–4.45).

Together, these trajectories represent distinct magnitudes and combinations of short-term change across clinical and psychological outcomes.

### 3.3. Distribution of Recovery Trajectories by Treatment Group

**Figure 1:**
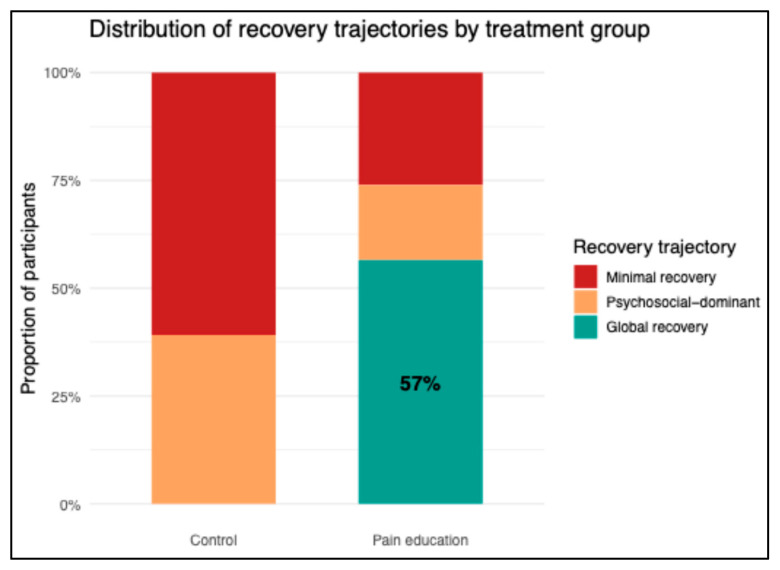
Distribution of short-term recovery trajectories by treatment group. Participants in the control group were exclusively classified within the minimal recovery and psychosocial-dominant recovery trajectories, with none achieving global recovery. In contrast, a higher proportion of participants in the pain education group were classified as being in the global recovery trajectory.

The distribution of recovery patterns differed between groups (Table 3, Figure 1). In the control group, participants were classified exclusively into the minimal recovery (28/46; 61%) and psychosocial-dominant recovery trajectories (18/46; 39%), with no participants achieving global recovery. In contrast, in the pain education group, more than half of the participants were classified as global recovery (26/46; 57%), while substantially fewer were classified as minimal recovery (12/46; 26%) or psychosocial-dominant recovery (8/46; 17%).

The association between treatment allocation and recovery trajectory was statistically large and highly significant (χ^2^(2) = 36.25, *p* < 0.001; Cramer’s V = 0.63). When recovery trajectories were dichotomized into global recovery versus all other patterns, global recovery occurred in 26 of 46 participants (57%) in the pain education group and none of the 46 participants in the control group (Fisher’s exact *p* < 0.001). Together, these findings indicate that pain education was associated with a marked redistribution of recovery trajectories toward the global recovery trajectory.

### 3.4. Sensitivity Analyses

Sensitivity analyses using multinomial logistic regression, adjusting for baseline pain, disability, age, sex, smoking status, and symptom duration, yielded results consistent with the primary analyses. The association between pain education and psychosocial-dominant recovery trajectory was not statistically significant (adjusted OR 1.61, 95% CI 0.41–6.29; *p* = 0.49). Estimation of the global recovery trajectory was limited by the absence of global recovery cases in the control group (Table S1).

## 4. Discussion

### 4.1. Summary of Findings

This secondary analysis identified three distinct short-term recovery trajectories following physiotherapy alone or physiotherapy combined with pain education in people with CLBP. These trajectories reflected different multidimensional changes across pain, disability, psychological well-being, and self-efficacy, rather than differences in a single outcome. The trajectories were labeled minimal recovery, Psychosocial-dominant recovery, and global recovery.

The distribution of recovery trajectories differed between treatment groups. More than half of the participants who received physiotherapy with pain education were classified within the global recovery trajectory, showing large and consistent improvements across all measured outcomes. In contrast, no participants in the physiotherapy-only group were classified within the global recovery trajectory. Participants in this group were classified only as minimal recovery or psychosocial-dominant trajectory.

A psychosocial-dominant recovery trajectory was observed in both groups, characterized by improvements in well-being and self-efficacy alongside relatively modest changes in pain and disability. After adjustment for baseline factors, this pattern was not clearly associated with treatment allocation. Overall, the findings suggest that adding pain education to the physiotherapy program was associated with a marked redistribution of short-term recovery patterns, particularly an increased representation of the global recovery trajectory.

### 4.2. Global Recovery Pattern

The global recovery trajectory was characterized by large improvements in multiple outcomes that occurred simultaneously (e.g., pain intensity, disability, psychological well-being, and self-efficacy), indicating coherent change across multiple outcome domains rather than isolated improvement in symptoms. This trajectory resulted from an exploratory and data-driven clustering approach. Thus, it describes an observed recovery pattern rather than a discrete, mechanistically defined recovery state. Global recovery was only observed in participants who received the pain education plus physiotherapy group, indicating a significant redistribution of recovery trajectories between treatment groups and movement towards broad and multidimensional improvement.

However, this observed difference in recovery distribution should not be interpreted as evidence that pain education alone fully accounts for the observed global recovery pattern. Although baseline characteristics were broadly comparable between groups, moderate imbalances were observed in symptom duration and smoking status. Sensitivity analyses adjusting for baseline pain, disability, age, sex, smoking status, and symptom duration yielded findings consistent with the primary analyses (Table S1). Nevertheless, the absence of global recovery cases in the control group limited the precision of adjusted effect estimates. Accordingly, these findings indicate an association between treatment allocation and recovery trajectory classification and should not be interpreted as evidence of a definitive causal effect.

The pattern of global recovery seen in this study is consistent with the current models of LBP that highlight the interdependence of pain, psychological processes, and coping capacity [2,4]. The simultaneous improvements in these outcomes could be explained by alterations in pain-related understandings and threat appraisals that may influence multiple domains of outcome at the same time, not just pain alone. In support of this interpretation, recent syntheses of pain neuroscience education report modest average effects on pain and disability but more consistent effects on psychosocial outcomes, and with substantial heterogeneity across individuals [1,17]. Taken together, this body of evidence suggests that there may be broad, multidimensional recovery in a subset of patients and that mean-based analyses may not adequately capture this recovery.

The physiotherapy intervention assigned to the control group was delivered in accordance with current clinical practice guidelines and reflects standard evidence-based care [1,2,3]. Although no participants in the physiotherapy-only group were classified within the global recovery trajectory, this finding should be interpreted with caution. It should not be interpreted as suggesting that physiotherapy alone is incapable of producing global recovery. Rather, within this sample and the six-week follow-up period, global recovery was observed only among participants who received additional pain education. Several methodological considerations may help explain this pattern. The sample size in this secondary analysis was modest and not specifically powered to detect differences in trajectory distribution, and the clustering approach was exploratory and data-driven. Moderate baseline imbalances were also observed, and although adjusted sensitivity analyses yielded consistent findings, residual confounding cannot be excluded. In addition, the follow-up period reflects short-term recovery only, and broader multidimensional improvements following physiotherapy alone may emerge over longer time horizons.

### 4.3. Psychosocial Recovery Pattern

The pattern of recovery in the psychosocial-dominant trajectory was characterized by large improvements in psychological well-being and self-efficacy with modest improvements in pain intensity and disability. This profile suggests a type of recovery where psychosocial adaptation improves more quickly than physical symptoms, rather than a partial or incomplete response to treatment. Importantly, this trajectory was found in both treatment groups, indicating that it is not specific to the addition of pain education.

After controlling for baseline characteristics, the analyses showed no clear association between allocation to the treatment arm and classification within the psychosocial-dominant recovery trajectory (see Supplementary Table S1). Consequently, the observed improvements in well-being and self-efficacy cannot be attributed solely to the pain education component of the intervention. More likely, these improvements reflect participants’ responsiveness to the physiotherapy intervention provided to both groups. All participants undertook a standardized physiotherapy regimen that adhered to guidelines and included advice to remain active, supervised exercise sessions, and repeated therapist–patient interactions. The structured nature of this rehabilitation program may have facilitated improvements in psychological well-being and self-efficacy, independent of the additional pain education material. Indeed, similar psychosocial-dominant response patterns have been observed in previous trajectory and subgroup studies, in which psychological improvements occurred despite relatively modest reductions in pain intensity or functional disability [24,26].

Clinically, this trajectory should not be interpreted as treatment failure. Rather, it may represent an intermediate or alternative form of recovery in which psychological adaptation precedes, or occurs independently of, symptomatic improvement. However, given the short follow-up period, it remains unclear whether psychosocial-dominant recovery is a stable trajectory, a transient response, or a precursor to later improvements in pain and disability. Longer-term follow-up is required to determine whether this trajectory translates into sustained functional recovery or reduced healthcare use over time.

### 4.4. Clinical Implications

The findings of this study indicate that short-term recovery following treatment for CLBP is heterogeneous, with patients exhibiting different recovery trajectories over the same intervention period. These differences are not apparent when outcomes are summarized using single outcomes or mean changes alone [23,24], which may obscure clinically meaningful variation in how patients respond to treatment.

The high proportion of participants classified within the global recovery trajectory in the pain education group should not be interpreted as evidence that pain education reliably produces comprehensive recovery at the individual level. Rather, the results suggest that some patients experience broad improvements across pain, disability, and psychosocial domains, while others demonstrate predominantly psychological improvements, with more modest changes in symptoms.

Importantly, improvements in psychological well-being and self-efficacy may represent meaningful clinical progress, even in the absence of large reductions in pain or disability. However, the present findings do not support changes in treatment selection, stratification, or clinical decision-making based on recovery trajectory membership. Instead, these results support a broader, multidimensional view of recovery and suggest that trajectory-based interpretation of outcomes may improve understanding of heterogeneous short-term responses to treatment in individuals with LBP.

### 4.5. Strengths and Limitations

One of the strengths of this study is that a multivariate, trajectory-based analytic approach was used to investigate the short-term recovery after intervention for people with CLBP. By incorporating changes across pain, disability, psychological well-being, and self-efficacy, this approach goes beyond single-outcome or mean-based summaries and offers a more comprehensive perspective on heterogeneous responses to recovery, a perspective increasingly advocated [42]. The use of data from an RCT provides additional internal validity, enhancing the interpretation of observed recovery trajectories as reflecting differences in response rather than systematic variation in intervention delivery. In addition, sensitivity analyses based on adjusted multinomial models were performed to assess the robustness of trajectory-group associations, accounting for statistical uncertainty.

However, several limitations should also be noted. Recovery trajectories in this study were determined using an exploratory, data-driven clustering approach and, as such, should be interpreted as descriptive rather than definitive classifications. The sample size was based on the primary RCT and was not specifically calculated for the exploratory clustering analyses performed in this secondary study. An additional limitation relates to the duration of follow-up. The identified recovery trajectories reflect patterns observed over a six-week period only. It remains unclear whether these trajectories represent sustained recovery states or short-term treatment responses, and their prognostic significance over longer follow-up is unknown. Future studies incorporating extended follow-up are required to evaluate the stability of multidimensional recovery patterns. Furthermore, the absence of global recovery cases in the control group led to quasi-complete separation in the adjusted multinomial models, limiting the precision and reliability of independent effect estimates for this trajectory.

## 5. Conclusions

This secondary analysis identified three different short-term recovery trajectories after physiotherapy with or without pain education for people with CLBP. Rather than reflecting change in a single outcome, these trajectories described parallel changes in pain intensity, disability, psychological well-being, and self-efficacy over the six weeks of intervention. Most of the participants who received physiotherapy with pain education were classified within a global recovery trajectory. In contrast, those who were assigned to physiotherapy alone were represented only within the minimal recovery and psychosocial-dominant trajectories. The psychosocial-dominant trajectory was observed in both groups and characterized improvements in psychological outcomes, with relatively small changes in pain and disability.

## Figures and Tables

**Table 1:** Baseline characteristics of participants by treatment group.

Variable	Level	Control (n = 46)	Pain Education (n = 46)
Age (years)		42.52 (13.32)	42.02 (8.24)
Sex	Female	35 (76.1)	25 (54.3)
	Male	11 (23.9)	21 (45.7)
Duration of low back pain (months)		35.85 (50.37)	20.93 (34.33)
VAS (0–10)		5.98 (1.71)	5.70 (1.66)
RMDQ (0–24)		15.02 (4.78)	12.65 (4.71)
WHO-5 (0–100)		54.41 (18.56)	53.48 (17.53)
GSE (10–40)		25.78 (6.40)	25.83 (5.61)
BMI (kg/m^2^)		26.96 (4.08)	27.76 (4.28)
Hours of sleep (per night)		6.33 (1.73)	6.74 (1.32)
Smoker	No	42 (91.3)	34 (73.9)
	Yes	4 (8.7)	12 (26.1)
Level of activity	Light	14 (30.4)	17 (37.0)
	Moderate	23 (50.0)	20 (43.5)
	Very active	9 (19.6)	9 (19.6)

Values are mean (SD) unless otherwise stated; categorical variables are presented as n (%). GSE = General Self-Efficacy Scale; WHO-5 = World Health Organization-5 Well-Being Index; RMDQ = Roland–Morris Disability Questionnaire; VAS = Visual Analogue Scale for pain; BMI = body mass index.

**Table 2:** Recovery trajectory clusters by outcome (mean change [SD] and 95% confidence intervals.

Outcomes	Minimal Recovery (n = 40)		Psychosocial-Dominant Recovery (n = 26)		Global Recovery (n = 26)	
	Mean (SD)	95% CI	Mean (SD)	95% CI	Mean (SD)	95% CI
VAS (0–10)	−0.83 (1.77)	−1.39, −0.26	−1.54 (1.58)	−2.18, −0.9	−4.00 (1.30)	−4.52, −3.48
RMDQ (0–24)	−1.58 (3.20)	−2.6, −0.55	−1.73 (2.16)	−2.6, −0.86	−9.77 (3.44)	−11.16, −8.38
WHO-5 (0–100)	2.50 (7.62)	0.06, 4.94	4.92 (7.03)	2.09, 7.76	24.19 (12.28)	19.23, 29.15
GSE (10–40)	0.75 (2.22)	0.04, 1.46	9.46 (2.75)	8.35, 10.57	2.69 (4.36)	0.93, 4.45

Values are mean change (SD) from baseline to post-intervention. Negative values for pain intensity and disability represent improvement; positive values for well-being and self-efficacy represent improvement. 95% CI = 95% confidence interval. GSE = General Self-Efficacy Scale; WHO-5 = World Health Organization-5 Well-Being Index; RMDQ = Roland–Morris Disability Questionnaire; VAS = Visual Analogue Scale for pain.

**Table 3:** Distribution of recovery trajectory by treatment group (n and %).

Trajectory	Control n (%)	Pain Education n (%)
Minimal recovery	28 (61)	12 (26)
Psychosocial-dominant recovery	18 (39)	8 (17)
Global recovery	0 (0.0)	26 (57)

Values are n (% of group). Percentages are calculated within the treatment group (columns sum to 100%). Minimal recovery = minimal change in pain, disability, well-being, and self-efficacy; Psychosocial-dominant recovery = modest symptom changes with large gains in self-efficacy and well-being; Global recovery = large, coherent improvements across all outcomes.

## Data Availability

This is a secondary analysis of a published randomized controlled trial. The data is available publicly. The R code used to perform the analyses was implemented in R software (version 4.5.2) and is available from the author upon reasonable request.

## Supplementary Materials

The following supporting information can be downloaded at https://www.mdpi.com/article/10.3390/jcm15062320/s1. Table S1: Multinomial logistic regression for recovery trajectories (reference: Minimal recovery trajectory).

## References

[1] Foster N.E., Anema J.R., Cherkin D., Chou R., Cohen S.P., Gross D.P., Ferreira P.H., Fritz J.M., Koes B.W., Peul W. (2018). Prevention and treatment of low back pain: Evidence, challenges, and promising directions. Lancet.

[2] Hartvigsen J., Hancock M.J., Kongsted A., Louw Q., Ferreira M.L., Genevay S., Hoy D., Karppinen J., Pransky G., Sieper J. (2018). What low back pain is and why we need to pay attention. Lancet.

[3] Aldera M., Alturkistany A., Al Rayes H., Rada G., Alsulaimany H.H., Alsobayel H.I., Alghamdi K., Awwad W., Alyamani O.A., Bedaiwi M. (2026). Saudi Clinical Practice Guideline for the Assessment and Management of Low Back Pain and Sciatica in Adults. J. Clin. Med..

[4] Lee H., Hübscher M., Moseley G.L., Kamper S.J., Traeger A.C., Mansell G., McAuley J.H. (2015). How does pain lead to disability? A systematic review and meta-analysis of mediation studies in people with back and neck pain. Pain.

[5] Vlaeyen J.W., Linton S.J. (2012). Fear-avoidance model of chronic musculoskeletal pain: 12 years on. Pain.

[6] Costa L.d.C.M., Maher C.G., Hancock M.J., McAuley J.H., Herbert R.D., Costa L.O. (2012). The prognosis of acute and persistent low-back pain: A meta-analysis. CMAJ.

[7] Qaseem A., Wilt T.J., McLean R.M., Forciea M.A., Denberg T.D., Barry M.J., Boyd C., Chow R.D., Fitterman N., Clinical Guidelines Committee of the American College of Physicians (2017). Noninvasive treatments for acute, subacute, and chronic low back pain: A clinical practice guideline from the American College of Physicians. Ann. Intern. Med..

[8] Gatchel R.J., Peng Y.B., Peters M.L., Fuchs P.N., Turk D.C. (2007). The biopsychosocial approach to chronic pain: Scientific advances and future directions. Psychol. Bull..

[9] McCracken L.M., Morley S. (2014). The psychological flexibility model: A basis for integration and progress in psychological approaches to chronic pain management. J. Pain.

[10] Moseley G.L., Butler D.S. (2015). The Explain Pain Handbook: Protectometer.

[11] Moseley G.L., Butler D.S. (2017). Explain Pain Supercharged.

[12] Sharma S., Jensen M.P., Moseley G.L., Abbott J.H. (2018). Pain education for patients with non-specific low back pain in Nepal: Protocol of a feasibility randomised clinical trial (PEN-LBP Trial). BMJ Open.

[13] Sidiq M., Muzaffar T., Janakiraman B., Masoodi S., Vasanthi R.K., Ramachandran A., Bansal N., Chahal A., Kashoo F.Z., Rizvi M.R. (2024). Effects of pain education on disability, pain, quality of life, and self-efficacy in chronic low back pain: A randomized controlled trial. PLoS ONE.

[14] Ryum T., Stiles T.C. (2023). Changes in pain catastrophizing, fear-avoidance beliefs, and pain self-efficacy mediate changes in pain intensity on disability in the treatment of chronic low back pain. Pain Rep..

[15] Jackson T., Wang Y., Wang Y., Fan H. (2014). Self-efficacy and chronic pain outcomes: A meta-analytic review. J. Pain.

[16] Linton S.J., Shaw W.S. (2011). Impact of psychological factors in the experience of pain. Phys. Ther..

[17] Cancela J.G., Vázquez O.C., Ledesma S.N., Pruimboom L. (2025). The effectiveness of pain neuroscience education in people with chronic non-specific low back pain: An umbrella review with meta-analysis. Ann. Phys. Rehabil. Med..

[18] Fleckenstein J., Floessel P., Engel T., Krempel L., Stoll J., Behrens M., Niederer D. (2022). Individualized exercise in chronic non-specific low back pain: A systematic review with meta-analysis on the effects of exercise alone or in combination with psychological interventions on pain and disability. J. Pain.

[19] Hayden J.A., Ellis J., Ogilvie R., Malmivaara A., van Tulder M.W. (2021). Exercise therapy for chronic low back pain. Cochrane Database Syst. Rev..

[20] Kent D.M., Rothwell P.M., Ioannidis J.P., Altman D.G., Hayward R.A. (2010). Assessing and reporting heterogeneity in treatment effects in clinical trials: A proposal. Trials.

[21] Lesko C.R., Henderson N.C., Varadhan R. (2018). Considerations when assessing heterogeneity of treatment effect in patient-centered outcomes research. J. Clin. Epidemiol..

[22] Varadhan R., Seeger J.D. (2013). Estimation and reporting of heterogeneity of treatment effects. Developing a Protocol for Observational Comparative Effectiveness Research: A User’s Guide.

[23] Kent P., Keating J.L., Leboeuf-Yde C. (2010). Research methods for subgrouping low back pain. BMC Med. Res. Methodol..

[24] Kongsted A., Kent P., Axen I., Downie A.S., Dunn K.M. (2016). What have we learned from ten years of trajectory research in low back pain?. BMC Musculoskelet. Disord..

[25] Aili K., Campbell P., Michaleff Z.A., Strauss V.Y., Jordan K.P., Bremander A., Croft P., Bergman S. (2021). Long-term trajectories of chronic musculoskeletal pain: A 21-year prospective cohort latent class analysis. Pain.

[26] Dunn K.M., Campbell P., Jordan K.P. (2013). Long-term trajectories of back pain: Cohort study with 7-year follow-up. BMJ Open.

[27] Kongsted A., Andersen C.H., Hansen M.M., Hestbaek L. (2016). Prediction of outcome in patients with low back pain–a prospective cohort study comparing clinicians’ predictions with those of the start back tool. Man. Ther..

[28] Alalawi A. (2026). Causal Mediation Analysis of the Effects of Pain Education on Disability and Pain Intensity in Individuals with Chronic Low Back Pain. J. Clin. Med..

[29] National Guideline Centre (UK) (2016). Low Back Pain and Sciatica in over 16s: Assessment and Management.

[30] Hawker G.A., Mian S., Kendzerska T., French M. (2011). Measures of adult pain: Visual analog scale for pain (vas pain), numeric rating scale for pain (nrs pain), mcgill pain questionnaire (mpq), short-form mcgill pain questionnaire (sf-mpq), chronic pain grade scale (cpgs), short form-36 bodily pain scale (sf-36 bps), and measure of intermittent and constant osteoarthritis pain (icoap). Arthritis Care Res..

[31] Roland M., Fairbank J. (2000). The Roland–Morris disability questionnaire and the Oswestry disability questionnaire. Spine.

[32] Topp C.W., Østergaard S.D., Søndergaard S., Bech P. (2015). The WHO-5 Well-Being Index: A systematic review of the literature. Psychother. Psychosom..

[33] Luszczynska A., Scholz U., Schwarzer R. (2005). The general self-efficacy scale: Multicultural validation studies. J. Psychol..

[34] Vlaeyen J.W., Linton S.J. (2000). Fear-avoidance and its consequences in chronic musculoskeletal pain: A state of the art. Pain.

[35] Jolliffe I.T., Cadima J. (2016). Principal component analysis: A review and recent developments. Philos. Trans. R. Soc. A Math. Phys. Eng. Sci..

[36] Ringnér M. (2008). What is principal component analysis?. Nat. Biotechnol..

[37] Costello A.B., Osborne J. (2005). Best practices in exploratory factor analysis: Four recommendations for getting the most from your analysis. Pract. Assess. Res. Eval..

[38] Jolliffe I. (2005). Principal component analysis. Encyclopedia of Statistics in Behavioral Science.

[39] Hastie T., Tibshirani R., Friedman J., Franklin J. (2005). The elements of statistical learning: Data mining, inference and prediction. Math. Intell..

[40] Kaufman L., Rousseeuw P.J. (2009). Finding Groups in Data: An Introduction to Cluster Analysis.

[41] Bilder C.R., Loughin T.M. (2024). Analysis of Categorical Data with R.

[42] Kent D.M., Paulus J.K., Van Klaveren D., D’Agostino R., Goodman S., Hayward R., Ioannidis J.P., Patrick-Lake B., Morton S., Pencina M. (2020). The predictive approaches to treatment effect heterogeneity (PATH) statement. Ann. Intern. Med..

