# Supplementary material for: Multidimensional Recovery Trajectories Following Physiotherapy with or Without Pain Education in People with Chronic Low Back Pain
Source: J Clin Med. 2026 Mar 18;15(6):2320. doi: 10.3390/jcm15062320 (PMC13027208; doi:10.3390/jcm15062320)
Supplement: Supplementary file 1 [file jcm-15-02320-s001.zip › jcm-4179045-supplementary.pdf]

Table S1: Multinomial logistic regression for recovery trajectories (reference: Minimal recovery trajectory).

For the psychosocial-dominant recovery vs minimal recovery, the adjusted odds ratio (OR) for the pain education group versus control was 1.61 (95% CI 0.41-6.29; p = 0.49), indicating no clear evidence that pain education changed the probability of being classified into the psychosocial-dominant trajectory rather than the minimal recovery trajectory after accounting for covariates. Within this comparision, higher baseline pain was the only covariate associated with trajectory allocation (OR 1.42, 95% CI 1.01-2.00; p = 0.043), whereas age, baseline disability, duration, sex, and smoking showed no meaningful associations.

For the global recovery vs minimal recovery, the model exhibited quasi-complete separation because no participants in the control group were classified as global recovery. Consequently, the adjusted OR for pain education vs control was not estimable and appears numerically extremely large, with p < 0.001 (Table S1). This reflects the same phenomenon seen in the raw data (57% vs 0% global recovery) rather than a precise effect size estimate. In this contrast, higher baseline disability (OR 1.62, 95% CI 1.17-2.25; p = 0.004) and higher baseline pain (OR 1.83, 95% CI 1.02-3.29; p = 0.044) were associated with greater odds of global recovery relative to minimal recovery, whereas age, duration, sex, and smoking were not clearly related.

Table S1. Multinomial logistic regression for recovery trajectories (reference: Minimal recovery trajectory).

Predictor	Adjusted OR	95% CI	p-value
**Psychosocial-dominant vs Minimal recovery**			
Age (years)	0.97	0.93-1.02	0.224
Baseline disability (RMDQ)	0.99	0.87-1.13	0.883
Baseline pain (VAS)	**1.42**	**1.01-2.00**	**0.043**
Duration of low back pain (months)	1.00	0.99-1.02	0.501
Sex: Males	0.57	0.14-2.33	0.431
Smoking Status: None-smokers	1.22	0.20-7.43	0.831
Treatment: Pain education vs Control	1.61	0.41-6.29	0.494
**Global recovery vs Minimal recovery**			
Age (years)	0.94	0.84-1.06	0.303
Baseline disability (RMDQ)	1.62	1.17-2.25	0.004
Baseline pain (VAS)	1.83	1.02-3.29	0.044

Table S1. Multinomial logistic regression for recovery trajectories (reference: Minimal recovery trajectory).

Predictor	Adjusted OR	95% CI	p-value
Duration of low back pain (months)	0.99	0.97-1.02	0.685
Sex: Males	0.75	0.10-5.67	0.780
Smoking Status: None-smokers	7.60	0.58-99.68	0.123
Treatment: Pain education vs Control	Not estimable *	Not estimable *	<0.001

Odds ratios (ORs) are from a multinomial logistic regression model with recovery trajectory as the outcome (reference category: minimal recovery trajectory) and predictors including treatment group, baseline pain (VAS), baseline disability (RMDQ), age, sex, smoking status, and duration of low back pain. ORs >1 indicate higher odds of the specified recovery trajectory relative to minimal recovery trajectory.

* For the contrast "Global recovery vs Minimal recovery, Treatment: Pain education vs Control", the model showed quasi-complete separation because no participants in the control group were classified as global recovery; the adjusted OR is therefore not estimable and appears numerically extremely large. The corresponding p-value (<0.001) reflects the strong association observed in the raw data.
